# Sodium-glucose cotransporter-2 inhibitors improve clinical outcomes in patients with type 2 diabetes mellitus undergoing anthracycline-containing chemotherapy: an emulated target trial using nationwide cohort data in South Korea

**DOI:** 10.1038/s41598-023-48678-1

**Published:** 2023-12-08

**Authors:** Hui-Jeong Hwang, Minji Kim, Ji Eun Jun, Dong Keon Yon

**Affiliations:** 1 Department of Cardiology, Kyung Hee University College of Medicine, Kyung Hee University Hospital at Gangdong, Seoul, 05278 Republic of Korea; 2 Center for Digital Health, Medical Science Research Institute, Kyung Hee University Medical Center, Kyung Hee University College of Medicine, Seoul, 02447 Republic of Korea; 3 Department of Regulatory Science, Kyung Hee University, Seoul, Republic of Korea; 4 Department of Endocrinology and Metabolism, Kyung Hee University Hospital at Gangdong, Kyung Hee University School of Medicine, Seoul, Republic of Korea

**Keywords:** Cardiology, Endocrinology, Oncology

## Abstract

Novel hypoglycemic agents, sodium-glucose cotransporter 2 inhibitors (SGLT2i), have shown protective effects against anthracycline (AC)-induced cardiotoxicity and exhibit partial anticancer effects in animal models. However, clinical evidence for this is scarce. This study aimed to evaluate whether SGLT2i improve the clinical outcomes of patients with type 2 diabetes mellitus (T2DM) undergoing AC-containing chemotherapy. A total of 81,572 patients who underwent AC chemotherapy between 2014 and 2021 were recruited from a nationwide Korean cohort. Patients were classified into three groups: patients with T2DM taking SGLT2i (n = 780) and other hypoglycemic agents excluding SGLT2i (non-SGLT2i; n = 3,455) during AC chemotherapy, and the non-DM group (n = 77,337). The clinical outcome was a composite of heart failure hospitalization, acute myocardial infarction, ischemic stroke, and death. After propensity score matching, 779 SGLT2i users were compared with 7800 non-DM patients and 2,337 non-SGLT2i users. The SGLT2i group had better composite outcomes compared with the non-DM group (adjusted hazard ratio [HR] = 0.35, 95% confidence interval [95% CI] = 0.25–0.51) and compared with the non-SGLT2i group (adjusted HR = 0.47, 95% CI = 0.32–0.69). In conclusion, SGLT2i may contribute to improving clinical outcomes in patients with T2DM undergoing AC-containing chemotherapy, through an emulated target trial using Korean nationwide cohort data.

## Introduction

As anticancer therapies advanced over the past decades, cancer-related mortality has decreased but the risk of cancer therapy-related cardiotoxicity has become an emerging concern in cancer survivors^1,2^. In particular, anthracyclines (ACs) are effective chemotherapeutic agents for the treatment of several solid organ cancers and hematologic malignancies but are well known to cause dose-dependent cardiac toxicity^2^.

Previous experimental and clinical studies demonstrated that sodium-glucose cotransporter 2 inhibitors (SGLT2i) decrease inflammatory pathways^3,4^ and improve endothelial function^4^ and metabolic pathways^5,6^. Furthermore, the use of SGLT2i in patients with type 2 diabetes mellitus (T2DM) was associated with reduced arrhythmia^7^, atherosclerosis^8^, and major adverse cardiovascular (CV) events, including acute myocardial infarction (AMI)^9^, heart failure (HF)^10,11^, and CV death^10,11^. Similarly, in doxorubicin (DOX)-treated experimental models, SGLT2i reduced cardiotoxicity by reducing inflammatory cytokine expression, myocardial fibrosis, and apoptosis^12–15^. Recently, a retrospective study showed that the use of SGLT2i in patients with diabetes mellitus (DM) undergoing AC chemotherapy decreased CV events and mortality compared to those in non-SGLT2i users^16^. However, considering that the previous research was conducted with a small sample size, evidence for this is still limited. Thus, in this study, we investigated whether using SGLT2i during AC chemotherapy in patients with T2DM improves clinical outcomes compared with: (1) patients without DM and (2) patients with T2DM who are taking other oral hypoglycemic agents (OHA) excluding SGLT2i (non-SGLT2i), using the Korean nationwide cohort.

## Methods

### Data sources, study population, and design

This study used the nationwide claims data from the Health Insurance Review and Assessment (HIRA) Service database of South Korea. It provides demographic and diagnostic data based on the International Classification of Disease-10th Revision-Clinical Modification diagnostic codes, electronic data interchange procedure codes, and mortality information for the entire Korean population.

**Figure 1:**
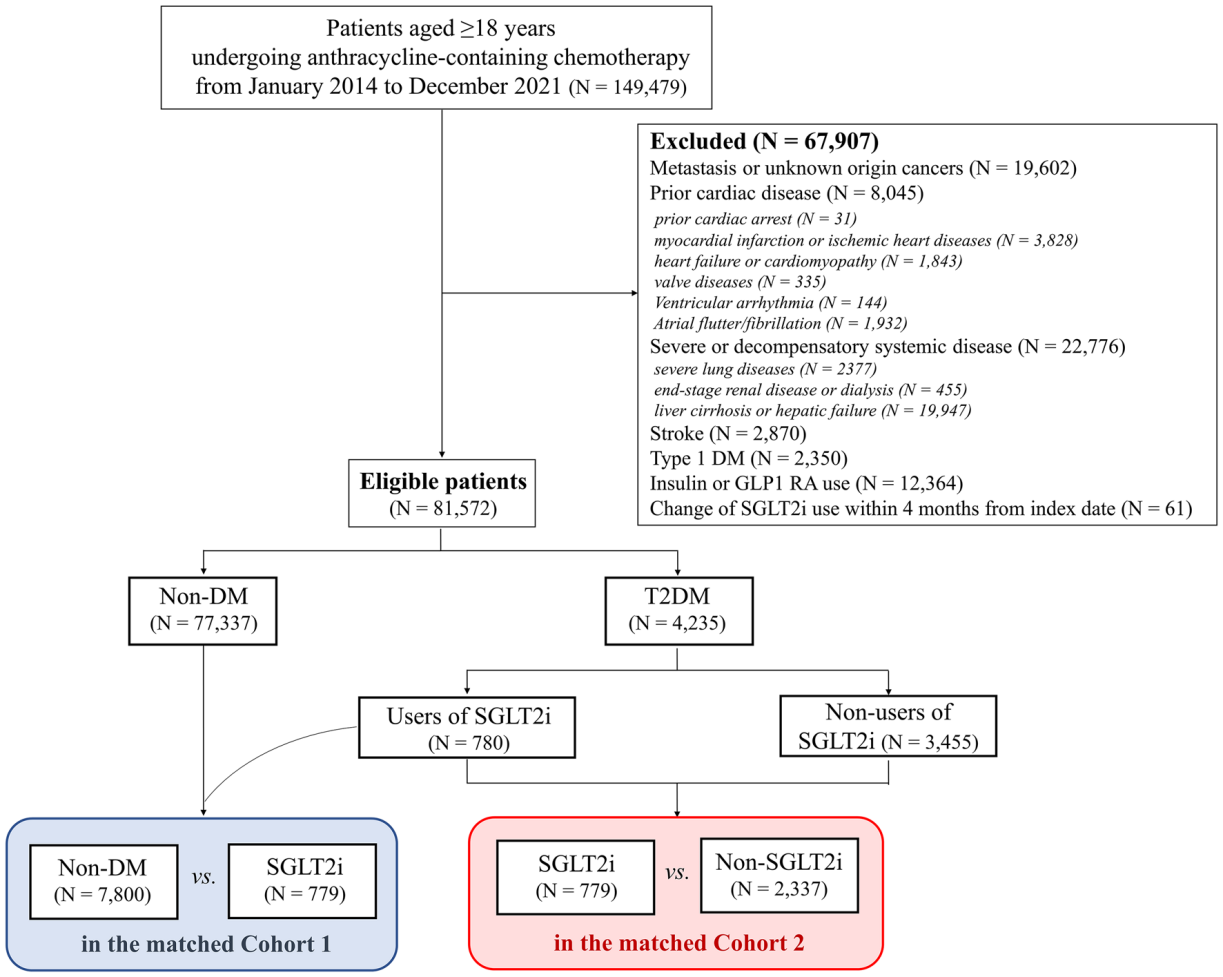
Study flow chart. DM—diabetes mellitus; GLP1 RA—glucagon-like peptide-1 receptor agonist; SGLT2i—sodium-glucose cotransporter-2 inhibitor.

A detailed flow chart of the study is shown in Fig. 1. Patients aged ≥ 18 years who were newly diagnosed with cancer and underwent AC-containing chemotherapy between January 2014 and December 2021 were recruited. The index date was defined as the date on which the ACs were first administered.

Exclusion criteria were as follows: patients diagnosed with cancer in 2013 or earlier; patients with metastasis or malignancy on other sites on the index date; patients with preexisting significant cardiac diseases, including cardiac arrest, myocardial infarction or ischemic heart disease, HF or cardiomyopathy, cardiac valve diseases, and atrial and ventricular arrhythmias; patients with a history of severe or decompensated systemic diseases, including severe lung diseases, end-stage renal diseases or dialysis, and liver cirrhosis or hepatic failure; patients with preexisting stroke; patients with type 1 DM; users of insulin or glucagon-like peptide-1 receptor agonists; and patients with change of SGLT2i within four months from the index date.

The patients were classified into three groups: those with and without SGLT2i use at the date of AC chemotherapy initiation among patients with T2DM (SGLT2i and non-SGLT2i groups) and those without DM (non-DM group). Two propensity score-matched cohorts were constructed to compare the clinical outcomes: (1) between the non-DM and SGLT2i groups in cohort 1 and (2) between the SGLT2i and non-SGLT2i groups in cohort 2. Detailed definitions of the diagnostic codes for inclusion and exclusion, comorbidities (including T2DM), and other chemotherapy information are described in Supplementary Table S1. High-dose AC was defined as > four cycles of DOX or a cumulative dose of > 240 mg/m^2^ and > five cycles of epirubicin or a cumulative dose of > 300 mg/m^2^ (equivalent dose of DOX as a reference)^17^.

This study was conducted according to the guidelines of the Declaration of Helsinki and approved by the Ethics Committee of the Kyung Hee University Hospital at Gangdong (KHNMC 2022–10-045). The requirement for informed consent was waived by the ethics committee of the Kyung Hee University Hospital at Gangdong because data from the HIRA Service database were anonymized and de-identified.

### Clinical outcomes

The following clinical outcomes were assessed: HF hospitalization, AMI, ischemic stroke, death, and the composite outcome of HF hospitalization, AMI, ischemic stroke, and death. Detailed definitions of the outcomes are provided in Supplementary Table S1. The patients were followed-up for outcome assessment until February 2022.

### Sample size calculation

Based on previous research and literature reviews^16,18^, we initially calculated a statistical power of 75% for each group to detect a 60% reduction in CV event incidence through SGLT2i use among patients with T2DM undergoing AC-containing chemotherapy, at a 5% significance level with a 1:2 ratio. The enrollment required would be 619 SGLT2i users and 1,238 non-SGLT2i users. Considering the missing values in this dataset, a sample size of 780 SGLT2i users and 3,455 non-SGLT2i users was deemed appropriate.

### Statistical analysis

Propensity score matching was performed to balance the baseline clinical characteristics between the two groups; in cohort 1: covariates included age, sex, indexed year, history of hypertension, dyslipidemia, coronary artery disease, use of antithrombotic agents, statins, renin-angiotensin-system (RAS) inhibitors, and beta-blockers, and cancer types; in cohort 2: covariates included T2DM duration and total numbers of distinct OHA classes along with covariates used in cohort 1.

Each propensity score-matched model used a greedy nearest-neighbor algorithm. The matching rates were determined based on the sample size between the groups as follows: 1:10 ratio between the SGLT2i and non-DM groups and 1:3 ratio between the SGLT2i and non-SGLT2i groups. A standardized mean difference ≤ 0.1 for a covariate was defined as well balanced. The crude event numbers and incidence rates of clinical outcomes were calculated for each propensity score-matched population. The incidence rate was expressed as per 100 person-years of follow-up. Clinical outcomes were compared using Cox proportional hazards models and presented as crude and adjusted hazard ratios (HRs) and 95% confidence intervals (95% CIs). The covariates used for adjustment were consistent with those used in each propensity score-matched model. For the sensitivity test, the 1-year composite outcomes were compared to each propensity score-matched cohort. Statistical analyses were performed using SAS version 9.4 (SAS Institute Inc., Cary, NC, USA) and R version 3.5. Statistical significance was set at a two-sided *p* < 0.05.

## Results

### Clinical characteristics

A total of 81,572 patients who initiated AC chemotherapy were recruited (Fig. 1). The non-DM, SGLT2i, non-SGLT2i groups consists of 77,337 (94.8%), 780 (1.0%), and 3,455 (4.2%) patients, respectively. The clinical characteristics of each group are presented in Table 1. The non-DM, SGLT2i, non-SGLT2i groups were 52 ± 12 years (men, 20%), 56 ± 10 years (men, 29%), and 62 ± 11 years (men, 37%), respectively.

**Table 1:** Baseline clinical characteristics in the overall study population.

Characteristics	Non-DM	T2DM with and without SGLT2i		
		Total	SGLT2i	Non-SGLT2i
**Subjects, n**	77,337	4,235	780	3,455
**Age, years (mean ± SD)**	52 ± 12	61 ± 11	56 ± 10	62 ± 11
**Sex, n (%)**				
Men	15,657 (20)	1,486 (35)	223 (29)	1,263 (37)
Women	61,680 (80)	2,749 (65)	557 (71)	2,192 (63)
**DM duration, n (%)**				
< 1 year	NA	1,130 (27)	173 (22)	957 (28)
1 to 5 years	NA	1,120 (26)	220 (28)	900 (26)
≥ 5 years	NA	1,985 (47)	387 (50)	1,598 (46)
**DM medications, n (%)**				
SGLT2i	NA	780 (18)	780 (100)	0 (0)
Metformin	NA	4,077 (96)	760 (97)	3317 (96)
Sulfonylureas	NA	2,425 (57)	545 (18)	1880 (62)
Glinides	NA	85 (2)	15 (2)	70 (2)
Thiazolidinediones	NA	85 (2)	15 (3)	70 (2)
DPP4i	NA	3,174 (75)	621 (80)	2,553 (74)
α- glucosidase inhibitors	NA	365 (9)	85 (11)	280 (8)
**Distinct OHA class No**				
≤ 2	NA	1,941 (46)	98 (13)	1,843 (53)
≥ 3	NA	2,294 (54)	682 (87)	1,612 (47)
**Index year, n (%)**				
2014–2016	28,890 (37)	1,162 (27)	182 (23)	980 (28)
2017–2019	29,117 (38)	1,566 (37)	293 (38)	1,273 (37)
2020–2021	19,330 (25)	1,507 (36)	305 (39)	1,202 (35)
**Medical history, n (%)**				
Hypertension	13,590 (18)	2,156 (51)	375 (48)	1,781 (52)
Dyslipidemia	11,562 (15)	1,728 (41)	372 (48)	1,356 (39)
Coronary artery disease	1027 (1)	143 (3)	28 (4)	115 (3)
**Medications, n (%)**				
Antithrombotic agents	6,503 (8)	1,052 (25)	186 (24)	866 (25)
Statins	10,392 (13)	2,362 (56)	515 (66)	1,847 (53)
RAS inhibitors	10,997 (14)	1,943 (46)	367 (47)	1,576 (46)
Beta-blockers	4815 (6)	511 (12)	87 (11)	424 (12)
**Cancer type, n (%)**				
Lymphoma	10,221 (13)	614 (15)	97 (12)	517 (15)
Breast	51,045 (66)	2,096 (49)	473 (61)	1,623 (47)
Genitourinary	4,580 (6)	421 (10)	55 (7)	366 (11)
Other cancers	12,010 (16)	1,126 (27)	160 (21)	966 (28)
**Chemotherapy, n (%)**				
AC classes				
*Doxorubicin*	74,120 (95)	3,981 (94)	752 (96)	3,229 (93)
*Epirubicin*	2,987 (1)	248 (5)	28 (4)	220 (6)
*Doxorubicin + Epirubicin*	230 (4)	6 (1)	0 (0)	6 (1)
High-dose ACs	21,640 (28)	1,346 (32)	208 (27)	1,138 (33)
Alkylating agents	3,824 (5)	140 (3)	21 (3)	119 (3)
Antimicrotubule agents	34,372 (44)	1,383 (33)	306 (39)	1,077 (31)
HER2 inhibitors	11,730 (15)	474 (11)	91 (12)	383 (11)
VEGF-targeting agents	2,892 (4)	224 (5)	27 (3)	197 (6)

*AC* anthracycline; *DPP4i* dipeptidyl peptidase-4 inhibitors; *HER* human epidermal growth factor receptor; *NA* not applicable; *No* number; *OHA* oral hypoglycemic agent; *RAS* renin-angiotensin system; *SD* standard deviation; *SGLT2i* sodium-glucose cotransporter-2 inhibitors; *T2DM* type 2 diabetes mellitus; *VEGF* vascular endothelial growth factor.

After propensity score matching, the non-DM, SGLT2i, and non-SGLT2i groups included 7,800, 779, and 2,337, respectively (Table 2). The covariates in each propensity score model were generally well balanced.

**Table 2:** Covariates for propensity score-matched cohorts.

Covariates	Non-DM versus SGLT2i (cohort 1)			Non-SGLT2i versus SGLT2i (cohort 2)		
	Non-DM	SGLT2i	SMD	Non-SGLT2i	SGLT2i	SMD
Subjects, n	7,800	779		2,337	779	
Age, years (mean ± SD)	58 ± 11	56 ± 10	− 0.1649	56 ± 10	56 ± 10	0.0371
Men, n (%)	2,362 (30)	223 (29)	0.0363	633 (27)	223 (29)	− 0.0343
T2DM duration, n (%)						
< 1 year	–	–	–	585 (25)	173 (22)	0.0730
1 to 5 years	–	–		616 (26)	220 (28)	
≥ 5 years	–	–		1,139 (49)	387 (50)	
Distinct OHA class No, n (%)						
≤ 2	–	–	–	299 (13)	98 (13)	0.006
≥ 3	–	–		2,038 (87)	681 (87)	
Index year, n (%)						
2014–2016	2,004 (26)	182 (23)	0.0584	564 (24)	182 (23)	0.0227
2017–2019	2,801 (36)	293 (38)		884 (38)	293 (38)	
2020–2021	2,995 (38)	305 (39)		892 (38)	305 (39)	
Medical history, n (%)						
Hypertension	4,121 (53)	375 (48)	-0.0940	1,078 (46)	375 (48)	0.0403
Dyslipidemia	3678 (47)	372 (48)	0.0094	1,106 (47)	372 (48)	0.0069
Coronary artery disease	272 (3)	28 (4)	0.0058	46 (2)	28 (4)	0.0990
Medications, n (%)						
Antithrombotic agents	1,968 (25)	186 (24)	− 0.0315	495 (21)	186 (24)	0.0656
Statins	5,302 (68)	515 (66)	− 0.0424	1,504 (64)	515 (66)	0.0368
RAS inhibitors	3,786 (49)	367 (47)	− 0.0286	1,032 (44)	367 (47)	0.0593
Beta-blockers	988 (13)	87 (11)	− 0.0503	256 (11)	87 (11)	0.0027
Cancer type, n (%)						
Lymphoma	4,653 (60)	473 (61)	0.0245	276 (12)	97 (12)	0.0197
Breast	4,653 (60)	473 (61)	0.0191	1,465 (63)	473 (61)	-0.0405
Genitourinary	580 (7)	55 (7)	− 0.0145	167 (7)	55 (7)	-0.0033
Other cancers	1,699 (22)	160 (21)	− 0.0304	442 (19)	160 (21)	0.0409

*RAS* renin-angiotensin system; *SD* standard deviation; *SGLT2i* sodium-glucose cotransporter-2 inhibitor; *SMD* standardized mean difference; *OHA* oral hypoglycemic agent; *No* number; *T2DM* type 2 diabetes mellitus.

An SMD of < 0.1 indicates no major imbalance.

All SMD values were < 0.1 in each propensity score-matched cohort except for age in cohort 1 (SMD = 0.1649).

There was no significant difference in the use of chemotherapy, including high-dose ACs, alkylating agents, antimicrobial agents, human epidermal growth factor receptor 2 inhibitors, and vascular endothelial growth factor-targeting agents, between the two groups in each propensity score-matched cohort (Supplementary Table S2).

### Clinical outcomes

The mean follow-up duration for the clinical outcomes was 3.4 ± 2.3 years. In matched cohort 1, the incidence rate of the composite outcome was 3.95 per 100 person-years in the non-DM group and 1.52 per 100 person-years in the SGLT2i group (Table 3). After adjusting by covariates, the SGLT2i group had better composite outcome (adjust HR = 0.35, 95% CI = 0.25 – 0.51), ischemic stroke (adjust HR = 0.30, 95% CI = 0.09 – 0.96), and death (adjust HR = 0.33, 95% CI = 0.23 – 0.49) compared with the non-DM group. In matched cohort 2, the incidence rate of the composite outcome was 2.91 per 100 person-years in the non-SGLT2i group and 1.52 per 100 person-years in the SGLT2i group. After adjusting by covariates, the SGLT2i group had better composite outcome (adjust HR = 0.47, 95% CI = 0.32 – 0.69) and death (adjust HR = 0.42, 95% CI = 0.28 – 0.63) compared with the non-SGLT2i group.

**Table 3:** Crude event number and incidence rate and hazard ratios for clinical outcomes in the propensity score-matched cohorts.

Clinical outcomes	Non-DM versus SGLT2i (cohort 1)		Non-SGLT2i versus SGLT2i (cohort 2)	
	Non-DM	SGLT2i	Non-SGLT2i	SGLT2i
Composite outcome = HF hospitalization + AMI + Ischemic stroke + Death				
Event (incidence rate*)	891 (3.95)	31 (1.52)	182 (2.91)	32 (1.52)
Crude HR (95% CI)	1.0 (ref)	**0.36 (0.25 – 0.52)**	1.0 (ref)	**0.51 (0.35 – 0.75)**
Adjusted HR (95% CI)	1.0 (ref)	**0.35 (0.25 – 0.51)**	1.0 (ref)	**0.47 (0.32 – 0.69)**
HF hospitalization				
Event (incidence rate*)	60 (0.26)	2 (0.10)	6 (0.10)	2 (0.10)
Crude HR (95% CI)	1.0 (ref)	0.37 (0.09 – 1.52)	1.0 (ref)	1.08 (0.22 – 5.40)
Adjusted HR (95% CI)	1.0 (ref)	0.35 (0.08 – 1.43)	1.0 (ref)	2.04 (0.40 – 12.22)
AMI				
Event (incidence rate*)	11 (0.05)	1 (0.05)	5 (0.08)	1 (0.05)
Crude HR (95% CI)	1.0 (ref)	0.95 (0.12 – 7.40)	1.0 (ref)	0.60 (0.07 – 5.11)
Adjusted HR (95% CI)	1.0 (ref)	2.07 (0.15 – 28.26)	1.0 (ref)	1.20 (0.10 – 14.85)
Ischemic stroke				
Event (incidence rate*)	117 (0.52)	3 (0.15)	4 (0.06)	3 (0.15)
Crude HR (95% CI)	1.0 (ref)	**0.28 (0.09 – 0.87)**	1.0 (ref)	2.18 (0.49 – 9.74)
Adjusted HR (95% CI)	1.0 (ref)	**0.30 (0.09 – 0.96)**	1.0 (ref)	2.42 (0.50 – 11.81)
Death				
Event (incidence rate*)	770 (3.37)	26 (1.26)	171 (2.72)	26 (1.26)
Crude HR (95% CI)	1.0 (ref)	**0.35 (0.24 – 0.52)**	1.0 (ref)	**0.45 (0.30 – 0.69)**
Adjusted HR (95% CI)	1.0 (ref)	**0.33 (0.23 – 0.49)**	1.0 (ref)	**0.42 (0.28 – 0.63)**

Significant are in value [bold].

*AMI* acute myocardial infarction; *CI* confidence interval; *DM* diabetes mellitus; *HF* heart failure; *HR* hazard ratio; *SGLT2i* sodium-glucose cotransporter-2 inhibitor.

Numbers in boldface indicate significant differences (*p* < 0.05).

*Per 100 person-years.

In the 1-year composite outcome, the SGLT2i group had better composite outcomes compared with the non-DM group (adjust HR = 0.41, 95% CI = 0.26 – 0.66) and the non-SGLT2i group (adjust HR = 0.40, 95% CI = 0.25 – 0.67) (Supplementary Table S3).

## Discussion

**Figure 2:**
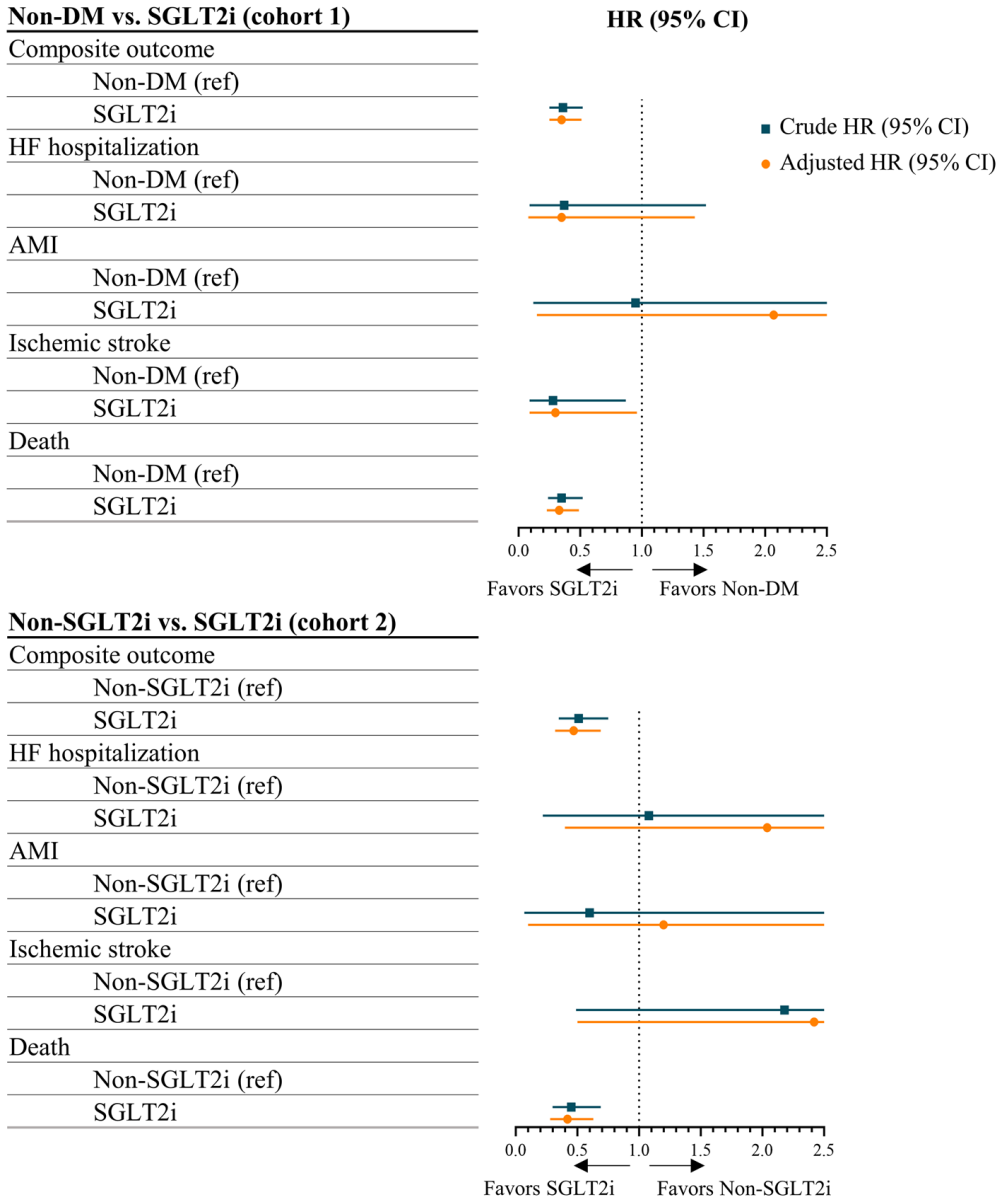
Crude and adjusted hazard ratios for clinical outcomes in the propensity score-matched cohorts. AMI—acute myocardial infarction; CI—confidence interval; DM—diabetes mellitus; HF—heart failure; HR—hazard ratio; SGLT2i—sodium-glucose cotransporter-2 inhibitor.

DM is a risk factor for AC-induced cardiotoxicity^19,20^, and hypoglycemic SGLT2i have been proposed as potential cardioprotective agents against AC toxicity in in vitro and in vivo studies^12–15,21^. This study on cancer patients undergoing AC-containing chemotherapy found that SGLT2i users with T2DM experienced better clinical outcomes than non-DM patients or non-SGLT2i users with T2DM (Fig. 2).

### AC-induced cardiotoxicity and DM as a risk factor

ACs are potent anticancer agents that are widely used in patients with breast cancer, bladder cancer, sarcoma, lymphoma, and acute leukemia^17^. However, they are cardiotoxic in a dose-dependent manner and can cause cardiomyopathy even at low doses in patients with genetic susceptibility or several CV risks^22,23^. Currently, the most commonly accepted pathophysiologic mechanism of AC-induced cardiotoxicity is cardiomyocyte injury due to oxidative stress^22^. However, other mechanisms, including mitochondrial dysfunction, DNA damage, impaired iron handling, apoptosis, and autophagy dysfunction, have also been hypothesized^21,24–26^.

DM significantly increases the incidence of HF and atherosclerotic CV diseases such as AMI and ischemic stroke^21,27,28^. Furthermore, a recent meta-analysis assessing risk factors for AC-related cardiotoxicity found DM to be a significant risk factor (odds ratio [95% CI] = 1.74 [1.11 – 2.74])^20^. Therefore, patients with DM undergoing AC chemotherapy require more careful management.

### The cardioprotective effects and proposed mechanisms of SGLT2i in T2DM

Hyperglycemia leads to SGLT2 overexpression in human cardiomyocytes in vitro and in vivo, potentially resulting in systolic and diastolic dysfunction and, ultimately, heart failure, particularly in patients with DM^6^. Therefore, SGLT2i may modulate the intracellular glucose pathway in cardiomyocytes and are expected to have cardioprotective effects. Indeed, in a study by Marfella et al., SGLT2i blunted metabolic alterations resulting from hyperglycemia and insulin resistance in non-diabetic heart transplant recipients with DM^5^.

In animal and in vitro models, the use of SGLT2i improved endothelial function by reducing oxidative stress and inflammation^4^. Similarly, in patients with T2DM, the use of SGLT2i stabilized carotid plaque through anti-inflammatory action^8^; and decreased arrhythmic burden^7^, HF hospitalization, and CV death^9^ after AMI. Notably, the reduction in the inflammatory burden due to the use of SGLT2i occurred earlier than achieving control of hyperglycemia^29^. Therefore, it is suggested that the cardioprotective effects of SGLT2i may be glycemic-independent.

### The cardioprotective effects of SGLT2i against AC toxicity

In in vitro and in vivo models of DOX, SGLT2i-treated cardiomyocytes showed reduced pro-inflammatory and inflammatory cytokine production, fibrosis, and apoptosis^13,14,30,31^; improved cell viability^14^; restored mitochondrial dysfunction^30^; and enhanced cardiac energy production by improving adenosine triphosphate generation^32,33^. The cardioprotective effects of SGLT2i were consistent in both diabetic and non-diabetic models^21,30^. Moreover, SGLT2i-treated animal models showed less reduction in left ventricular ejection fraction (LVEF) and myocardial strain following DOX administration^13,15^. However, this experimental evidence does not guarantee the efficacy of SGLT2i in cancer patients undergoing AC-containing chemotherapy, necessitating further clinical studies. Gongora et al. showed that SGLT2i users had better CV outcomes than non-SGLT2i users in patients with DM undergoing AC chemotherapy (one event in the 32 SGLT2i group [3%] vs. 19 events in the 96 non-SGLT2i group [20%], *p* = 0.025)^16^. However, their study was a small retrospective study.

DM is a concurrent risk factor for CV disease^27,28^ and AC-induced cardiotoxicity^19,20^. Nevertheless, in our study, SGLT2i users with T2DM had a lower incidence of ischemic stroke compared to patients without DM. In addition, HF hospitalization was lower in SGTL2i users with T2DM (0.1 per 100 person-years in the SGLT2i group vs. 0.26 per 100 person-years in the non-DM group), although there was no statistically significant difference due to fewer event numbers. These findings suggest that SGLT2i may exert cardioprotective effects against AC toxicity. We also propose that this may be attributed to the anti-inflammatory action of SGLT2i.

### SGLT2i and decreased mortality in cancer patients

In our study, SGLT2i users with T2DM had lower mortality rates than non-DM patients or non-SGLT2i users with T2DM. In human prostate and lung cancer cells, SGLT2i reduced cellular proliferation and clonogenic survival by inhibiting glucose uptake and mitochondrial function^34^. A similar phenomenon has been observed in other cancer cell lines, including kidney^35^ and breast^36^. Animal models have consistently demonstrated that SGLT2i decrease the occurrence and growth of cancer cells^36–38^. The findings suggest that SGLT2i may attenuate the growth of SGLT2-expressing cancer cells by inhibiting glucose uptake and blocking several subsequent intracellular metabolic pathways. Furthermore, SGLT2i reduce body weight through glycosuria, consequently improving insulin resistance^38,39^. Reducing body weight has been reported to reduce the risk of certain cancers, including obesity-associated breast^40^ and colon^41^ cancers.

In a clinical study for patients with DM by Gongora et al., SGLT2i users had a lower mortality rate than non-SGLT2i users^16^. The authors suggested that this finding may be due to reduced CV events in SGLT2i users. However, the finding that SGLT2i users showed a lower mortality rate even in our study with fewer CV events cannot be explained simply by improved CV events following the use of SGLT2i, which can be partially due to the anticancer effects of SGLT2i.

### Study limitations

This study has some limitations. First, it was a claim-based cohort study. Therefore, asymptomatic HF, as assessed by LVEF decline, cannot be estimated as a clinical outcome. The HIRA Service database of South Korea also does not provide laboratory data, including creatinine clearance, blood glucose, and glycated hemoglobin levels, or demographic information, including cancer staging, body mass index, and smoking status. However, we supposed that general medical treatments, such as glycemic control, would be effectively managed, at least during adjuvant chemotherapy for primary cancers. Furthermore, several studies found that the protective effects of SGLT2i due to anti-inflammatory action are independent of glycemic control^29,42^. Additionally, propensity score matching in this study included T2DM duration and the total number of distinct OHA classes as covariates for adjusting T2DM severity and hyperglycemic status. Second, insurance coverage for SGLT2i has been available in South Korea since 2014. Therefore, patients diagnosed with cancer since 2014 were enrolled and the number in the SGLT2i group was relatively small. Third, we excluded patients with previous CV diseases, including HF; at-risk patients with highly uncontrolled hyperglycemia, such as insulin use or type 1 DM; and at-risk patients with vulnerability, such as metastatic cancers. This exclusion aimed to minimize the potential influence of unexpected confounding factors that could distort the comparison of clinical outcomes between the groups in claim data analysis. However, it may have resulted in fewer CV events, potentially leading to insignificant differences in HF hospitalization according to SGLT2i use. Fourth, we aimed to evaluate the protective effects of SGLT2i against AC toxicity. Thus, the persistence of SGLT2i was investigated for only four months from AC initiation. Therefore, the long-term outcomes according to the persistent use of SGLT2i were not estimated in this study. Fifth, since the study exclusively enrolled Korean patients with cancer, the effects of SGLT2i against AC toxicity remain inconclusive in non-East Asian populations. Further study using diverse global cohorts is necessary.

## Conclusions

Through an emulated target trial using Korean nationwide cohort data, SGLT2i may contribute to decreasing mortality and improving clinical outcomes in patients with T2DM undergoing AC-containing chemotherapy. Future randomized controlled trials, including laboratory data to elucidate underlying mechanisms, are necessary.

### Supplementary Information

Supplementary Information.

## Data Availability

All data analyzed during this study are included in this published article and its supplementary information files. Additionally, the datasets analyzed during the current study are not publicly available due to Korean legal restrictions, but they can be obtained from the corresponding author upon reasonable request.

## References

[1] Herrmann J, Lerman A, Sandhu NP, Villarraga HR, Mulvagh SL, Kohli M (2014). Evaluation and management of patients with heart disease and cancer: cardio-oncology. Mayo. Clin. Proc..

[2] Morelli MB (2022). Cardiotoxicity of anticancer drugs: Molecular mechanisms and strategies for cardioprotection. Front. Cardiovasc. Med..

[3] Sardu C (2023). SGLT2 breast expression could affect the cardiovascular performance in pre-menopausal women with fatty versus non fatty breast via over-inflammation and sirtuins' down regulation. Eur. J. Inter. Med..

[4] Salvatore T (2021). Cardiovascular benefits from gliflozins: Effects on endothelial function. Biomedicines..

[5] Marfella R (2022). Sodium/glucose cotransporter 2 (SGLT2) inhibitors improve cardiac function by reducing JunD expression in human diabetic hearts. Metabolism..

[6] Marfella R (2022). Sodium-glucose cotransporter-2 (SGLT2) expression in diabetic and non-diabetic failing human cardiomyocytes. Pharmacol. Res..

[7] Cesaro A (2022). In-hospital arrhythmic burden reduction in diabetic patients with acute myocardial infarction treated with SGLT2-inhibitors: Insights from the SGLT2-I AMI PROTECT study. Front. Cardiovasc. Med..

[8] D'Onofrio N (2021). Sodium-glucose co-transporter2 expression and inflammatory activity in diabetic atherosclerotic plaques: Effects of sodium-glucose co-transporter2 inhibitor treatment. Mol. Metab..

[9] Paolisso P (2023). Outcomes in diabetic patients treated with SGLT2-Inhibitors with acute myocardial infarction undergoing PCI: The SGLT2-I AMI PROTECT registry. Pharmacol. Res..

[10] Wiviott SD (2019). Dapagliflozin and cardiovascular outcomes in type 2 diabetes. N. Engl. J. Med..

[11] Zinman B (2015). Empagliflozin, cardiovascular outcomes, and mortality in type 2 diabetes. N. Engl. J. Med..

[12] Vuong JT, Stein-Merlob AF, Cheng RK, Yang EH (2022). Novel therapeutics for anthracycline induced cardiotoxicity. Front. Cardiovasc. Med..

[13] Sabatino J (2020). Empagliflozin prevents doxorubicin-induced myocardial dysfunction. Cardiovasc. Diabetol..

[14] Quagliariello V (2021). The SGLT-2 inhibitor empagliflozin improves myocardial strain, reduces cardiac fibrosis and pro-inflammatory cytokines in non-diabetic mice treated with doxorubicin. Cardiovasc. Diabetol..

[15] Barış V, Dinçsoy AB, Gedikli E, Zırh S, Müftüoğlu S, Erdem A (2021). Empagliflozin significantly prevents the doxorubicin-induced acute cardiotoxicity via non-antioxidant pathways. Cardiovasc. Toxicol..

[16] Gongora CA (2022). Sodium-glucose co-transporter-2 inhibitors and cardiac outcomes among patients treated with anthracyclines. JACC. Heart. Fail..

[17] Lyon AR (2022). 2022 ESC guidelines on cardio-oncology developed in collaboration with the european hematology association (EHA), the european society for therapeutic radiology and oncology (ESTRO) and the international cardio-oncology society (IC-OS). Eur. Heart. J..

[18] Abdel-Qadir H (2023). The association of sodium-glucose cotransporter 2 inhibitors with cardiovascular outcomes in anthracycline-treated patients with cancer. JACC CardioOncol..

[19] Zhang M, Yang H, Xu C, Jin F, Zheng A (2022). Risk factors for anthracycline-induced cardiotoxicity in breast cancer treatment: A meta-analysis. Front. Oncol..

[20] Qiu S (2021). Risk factors for anthracycline-induced cardiotoxicity. Front. Cardiovasc. Med..

[21] Russo M, Della Sala A, Tocchetti CG, Porporato PE, Ghigo A (2021). Metabolic aspects of anthracycline cardiotoxicity. Curr. Treat. Options. Oncol..

[22] Zamorano JL (2016). 2016 ESC Position Paper on cancer treatments and cardiovascular toxicity developed under the auspices of the ESC committee for practice guidelines: The task force for cancer treatments and cardiovascular toxicity of the European Society of Cardiology (ESC). Eur. Heart. J..

[23] Florescu DR, Nistor DE (2019). Therapy-induced cardiotoxicity in breast cancer patients: a well-known yet unresolved problem. Discov. Craiova..

[24] Ichikawa Y (2014). Cardiotoxicity of doxorubicin is mediated through mitochondrial iron accumulation. J. Clin. Invest..

[25] Henriksen PA (2018). Anthracycline cardiotoxicity: An update on mechanisms, monitoring and prevention. Heart..

[26] Koleini N, Kardami E (2017). Autophagy and mitophagy in the context of doxorubicin-induced cardiotoxicity. Oncotarget.

[27] Heidenreich PA (2022). 2022 AHA/ACC/HFSA guideline for the management of heart failure: A report of the american college of cardiology/American heart association joint committee on clinical practice guidelines. Circulation.

[28] Arnett DK (2019). 2019 ACC/AHA guideline on the primary prevention of cardiovascular disease: a report of the american college of cardiology/American heart association task force on clinical practice guidelines. Circulation.

[29] Paolisso P (2022). Infarct size, inflammatory burden, and admission hyperglycemia in diabetic patients with acute myocardial infarction treated with SGLT2-inhibitors: A multicenter international registry. Cardiovasc. Diabetol..

[30] Oh CM (2019). Cardioprotective potential of an SGLT2 inhibitor against doxorubicin-induced heart failure. Korean. Circ. J..

[31] Xu C (2018). Canagliflozin exerts anti-inflammatory effects by inhibiting intracellular glucose metabolism and promoting autophagy in immune cells. Biochem. Pharmacol..

[32] Verma S (2018). Empagliflozin increases cardiac energy production in diabetes: novel translational insights into the heart failure benefits of SGLT2 inhibitors. JACC. Basic. Transl. Sci..

[33] Hammoudi N (2017). Empagliflozin improves left ventricular diastolic dysfunction in a genetic model of type 2 diabetes. Cardiovasc. Drugs. Ther..

[34] Villani LA (2016). The diabetes medication Canagliflozin reduces cancer cell proliferation by inhibiting mitochondrial complex-I supported respiration. Mol. Metab..

[35] Kuang H, Liao L, Chen H, Kang Q, Shu X, Wang Y (2017). Therapeutic effect of sodium glucose co-transporter 2 inhibitor dapagliflozin on renal cell carcinoma. Med. Sci. Monit..

[36] Zhou J (2020). Sodium-glucose co-transporter-2 (SGLT-2) inhibition reduces glucose uptake to induce breast cancer cell growth arrest through AMPK/mTOR pathway. Biomed. Pharmacother..

[37] Shiba K (2018). Canagliflozin, an SGLT2 inhibitor, attenuates the development of hepatocellular carcinoma in a mouse model of human NASH. Sci. Rep..

[38] Nasiri AR, Rodriguesm MR, Li Z, Leitner BP, Perry RJ (2019). SGLT2 inhibition slows tumor growth in mice by reversing hyperinsulinemia. Cancer. Metab..

[39] Pereira MJ, Eriksson JW (2019). Emerging role of SGLT-2 inhibitors for the treatment of obesity. Drugs.

[40] Hardefeldt PJ, Penninkilampi R, Edirimanne S, Eslick GD (2018). Physical activity and weight loss reduce the risk of breast cancer: A meta-analysis of 139 prospective and retrospective studies. Clin. Breast. Cancer..

[41] Beeken RJ (2017). The impact of diet-induced weight loss on biomarkers for colorectal cancer: An exploratory study (INTERCEPT). Obes. Silver Spring..

[42] Marfella R (2023). SGLT-2 inhibitors and in-stent restenosis-related events after acute myocardial infarction: An observational study in patients with type 2 diabetes. BMC Med..

