# Supplementary material for: Sodium-glucose cotransporter-2 inhibitors improve clinical outcomes in patients with type 2 diabetes mellitus undergoing anthracycline-containing chemotherapy: an emulated target trial using nationwide cohort data in South Korea
Source: Sci Rep. 2023 Dec 8;13:21756. doi: 10.1038/s41598-023-48678-1 (PMC10709414; doi:10.1038/s41598-023-48678-1)
Supplement: Supplementary file 1 — Supplementary Information. [file 41598_2023_48678_MOESM1_ESM.pdf]

[Original Article]

Sodium-Glucose Cotransporter-2 Inhibitors Improve Clinical Outcomes in Patients With Type 2 Diabetes Mellitus Undergoing Anthracycline-Containing Chemotherapy: A Nationwide Cohort Study in South Korea

Hui-Jeong Hwang, Minji Kim, Ji Eun Jun, Dong Keon Yon

Supplementary Table S1. Definitions of diagnostic and excluded criteria, comorbidities, medications, and clinical outcomes.

	ICD-10-CM code*	Diagnostic definition
**Inclusion criteria**		
Anthracyclines		doxorubicin, epirubicin
Cancer type		
Lymphoma	C81-88	
Breast	C50	
Genitourinary	C51-C68	
Other cancers	C00-C26, C30-34, C37-38, C40-49, C69-76, C90-97	
**Excluded criteria**		
Metastasis or unknown-origin cancers	C39, C77-C80	
Prior cardiac arrest	I46	
Myocardial infarction or ischemic heart diseases	I21-I25	
Heart failure or cardiomyopathy	I50, I130, I132, I110, I42-I43	
Valvular heart diseases	I34, I35, I38, I39	
Ventricular arrhythmias	I470, I472, I490, I495, I498	
Atrial flutter/fibrillation	I48	
Severe lung diseases	J4402, J441, J4482, J4492, J4503, J4513, J46	
End-stage renal disease or dialysis	N181-185, Z49	
Liver cirrhosis or hepatic failure	K72, K74, K702-704, K711	
Stroke	I60-I64	
Type 1 DM	E10	
GLP1 RA user		exenatide, lixisenatide, dulaglutide

**DM diagnosis**		
Non-DM		Neither E10-14 diagnosis code nor hypoglycemic agent use (including insulin, GLP1 RA, and oral hypoglycemic agents)
Type 2 DM	E11, E13-14	diagnosis code plus oral hypoglycemic agents (biguanides, sulfonylureas, glinides, thiazolidinediones, dipeptidyl peptidase-4 inhibitors, alpha- glucosidase inhibitors, SGLT2i)
- *SGLT2i*		diagnosis code plus SGLT2i (empagliflozin, dapagliflozin, ipragliflozin, ertugliflozin)
- *Non-SGLT2i*		diagnosis code plus other oral hypoglycemic agents excluding SGLT2i
- *Oral hypoglycemic agents*		
biguanides		metformin
sulfonylureas		glibenclamide, gliclazide, glimepiride
glinides		nateglinide, repaglinide, mitiglinide
thiazolidinediones		pioglitazone, lobeglitazone
dipeptidyl peptidase-4 inhibitors		alogliptin, anagliptin, evogliptin, gemigliptin, linagliptin, saxagliptin, sitagliptin, teneligliptin, vildagliptin
alpha-glucosidase inhibitors		acarbose, voglibose, miglitol
**Comorbidities**		
Hypertension	I10, I11, I12, I13, I15	diagnostic code plus antihypertensive drugs (calcium channel blockers, RAS inhibitors, beta-blockers, thiazide and thiazide-like diuretics)

Dyslipidemia	E78	1) diagnostic code plus drugs including fibrates, omega-3 fatty acids, and nicotinic acid<br>2) statin user
Coronary artery disease	I20	
**Medications**		
Antithrombotic agents		aspirin, clopidogrel, cilostazol, ticlopidine, prasugrel, ticagrelor, ticlopidine, triflusal, warfarin, enoxaparin, apixaban, dabigatran, edoxaban, rivaroxaban
Statins		atorvastatin, rosuvastatin, simvastatin, fluvastatin, pitavastatin, pravastatin, cerivastatin
RAS inhibitors		alacepril, benazepril, captopril, cilazapril, delapril, enalapril, fosinopril, lisinopril, moexipril, ramipril, imidapril, perindopril, quinapril, spirapril, trandolapril, zofenopril, valsartan, losartan, azilsartan, candesartan, telmisartan, eprosartan, fimasartan, irbesartan, olmesartan
Beta-blockers		acebutolol, atenolol, betaxolol, bevantolol, bisoprolol, carteolol, carvedilol, celiprolol, cloranolol, metoprolol, nadolol, nebivolol, pindolol, propranolol, tertatolol
Alkylating agents		cyclophosphamide, ifosfamide
Antimicrotubule agents		docetaxel, paclitaxel
HER2 inhibitors		trastuzumab, pertuzumab, lapatinib, neratinib, dacomitinib, tucatinib

VEGF-targeting agents		aflibercept, bevacizumab, ramucirumab, axitinib, lenvatinib, vandetanib, cabozantinib, regorafenib, sunitinib, vandetanib, sorafenib, pazopanib
**Clinical outcomes**		
HF hospitalization	I50, I130, I132, I110, I420	diagnostic code plus hospitalization plus loop diuretics ≥2 (furosemide, torsemide)
Acute myocardial infarction	I21-I24	diagnostic code plus hospitalization plus<br>1) coronary angiography in acute myocardial infarction<br>2) PCI or CABG procedure
Ischemic stroke	I63-64	diagnostic code plus hospitalization plus brain MRI/CT

CABG, coronary artery bypass grafting; DM, diabetes mellitus; GLP1 RA, glucagon-like peptide-1 receptor agonist; HER, human epidermal growth factor receptor; HF, heart failure; ICD-10-CM, the International Classification of Disease-10th Revision-Clinical Modification; MRI/CT, magnetic resonance imaging or computed tomography; PCI, percutaneous coronary intervention; RAS, renin-angiotensin system; SGLT2i; sodium-glucose cotransporter-2 inhibitors; VEGF, vascular endothelial growth factor.
*Admission ≥1 or outpatient department ≥2.

Supplementary Table S2. Administered chemotherapy between groups in the propensity score-matched cohorts

Variables	Non-DM vs. SGLT2i (cohort 1)			Non-SGLT2i vs. SGLT2i (cohort 2)		
	**Non-DM**	**SGLT2i**	***SMD***	**Non-SGLT2i**	**SGLT2i**	***SMD***
**Chemotherapy, n (%)**						
High-dose ACs	2024 (25.95)	208 (26.67)	0.0163	614 (26.24)	208 (26.67)	0.0097
Alkylating agents	338 (4.33)	21 (2.69)	-0.0892	79 (3.38)	21 (2.69)	-0.0399
Antimicrotubule agents	2922 (37.46)	306 (39.23)	0.0364	939 (40.13)	306 (39.23)	-0.0183
HER2 inhibitors	927 (11.88)	91 (11.67)	-0.0068	291 (12.44)	91 (11.67)	-0.0236
VEGF-targeting agents	253 (3.24)	27 (3.46)	0.0121	103 (4.40)	27 (3.46)	-0.0484

AC, anthracycline; DM, diabetes mellitus; HER, human epidermal growth factor receptor; SGLT2i; sodium-glucose cotransporter-2 inhibitor; SMD, standardized mean difference; VEGF, vascular endothelial growth factor.

Supplementary Table S3. Hazard ratios for the 1-year composite outcome in the propensity score-matched cohorts

**Clinical outcomes**	**Non-DM vs. SGLT2i (cohort 1)**		**Non-SGLT2i vs. SGLT2i (cohort 2)**	
	Non-DM	SGLT2i	Non-SGLT2i	SGLT2i
**Composite outcome = HF hospitalization + AMI + Ischemic stroke + Death**				
Crude HR (95% CI)	1.0 (ref)	0.44 (0.27 – 0.70)	1.0 (ref)	0.44 (0.27 – 0.72)
Adjusted HR (95% CI)	1.0 (ref)	0.41 (0.26 – 0.66)	1.0 (ref)	0.40 (0.25 – 0.67)

AMI, acute myocardial infarction; CI, confidence interval; DM, diabetes mellitus; HF, heart failure; HR, hazard ratio; SGLT2i, sodium-glucose cotransporter-2 inhibitor.
